# Conversion of Glycerol to 3-Hydroxypropanoic Acid by Genetically Engineered *Bacillus subtilis*

**DOI:** 10.3389/fmicb.2017.00638

**Published:** 2017-04-18

**Authors:** Aida Kalantari, Tao Chen, Boyang Ji, Ivan A. Stancik, Vaishnavi Ravikumar, Damjan Franjevic, Claire Saulou-Bérion, Anne Goelzer, Ivan Mijakovic

**Affiliations:** ^1^Systems and Synthetic Biology Division, Department of Biology and Biological Engineering, Chalmers University of TechnologyGothenburg, Sweden; ^2^Chaire Agro-Biotechnologies Industrielles, AgroParisTechReims, France; ^3^Key Laboratory of Systems Bioengineering (Ministry of Education), Tianjin UniversityTianjin, China; ^4^Department of Biology, Faculty of Science, University of ZagrebZagreb, Croatia; ^5^UMR Génie et Microbiologie des Procédés Alimentaires (GMPA), AgroParisTech, Institut National de la Recherche Agronomique, Université Paris-SaclayThiverval Grignon, France; ^6^Mathématiques et Informatique Appliquuées du Génome à l’Environnement (MaIAGE), Institut National de la Recherche Agronomique, Université Paris-SaclayJouy-en-Josas, France; ^7^Novo Nordisk Foundation Center for Biosustainability, Technical University of DenmarkLyngby, Denmark

**Keywords:** 3-hydroxypropanoic acid, glycerol, *Bacillus subtilis*, metabolic engineering, glycerol kinase knock-out

## Abstract

3-Hydroxypropanoic acid (3-HP) is an important biomass-derivable platform chemical that can be converted into a number of industrially relevant compounds. There have been several attempts to produce 3-HP from renewable sources in cell factories, focusing mainly on *Escherichia coli, Klebsiella pneumoniae*, and *Saccharomyces cerevisiae*. Despite the significant progress made in this field, commercially exploitable large-scale production of 3-HP in microbial strains has still not been achieved. In this study, we investigated the potential of *Bacillus subtilis* as a microbial platform for bioconversion of glycerol into 3-HP. Our recombinant *B. subtilis* strains overexpress the two-step heterologous pathway containing glycerol dehydratase and aldehyde dehydrogenase from *K. pneumoniae*. Genetic engineering, driven by *in silico* optimization, and optimization of cultivation conditions resulted in a 3-HP titer of 10 g/L, in a standard batch cultivation. Our findings provide the first report of successful introduction of the biosynthetic pathway for conversion of glycerol into 3-HP in *B. subtilis*. With this relatively high titer in batch, and the robustness of *B. subtilis* in high density fermentation conditions, we expect that our production strains may constitute a solid basis for commercial production of 3-HP.

## Introduction

Concerns over usage of fossil fuels have led to the development of sustainable technologies to convert renewable materials into various compounds such as alcohols, acids, and chemicals (Hanai et al., 2007; Atsumi and Liao, 2008; Atsumi et al., 2008; McKenna and Nielsen, 2011; Borodina et al., 2015). One of the very important biomass-derivable platform chemicals is 3-hydroxypropanoic acid (3-HP; Choi et al., 2015). 3-HP can be used in the synthesis of bulk chemicals, such as the acrylic acid (patent: WO 2013192451 A1), and plastics (Valdehuesa et al., 2013). Production of 3-HP by chemical conversion is not desirable due to the high cost and toxic intermediates leading to environmental issues (Jiang et al., 2009; Kumar et al., 2013). Glycerol, a by-product of the biodiesel industry, is an attractive renewable starting material for production of 3-HP (Chen and Nielsen, 2016). The main bacterial pathway for the synthesis of 3-HP from glycerol consists of two enzymatic reactions. The first is catalyzed by a coenzyme B_12_-dependent glycerol dehydratase, which converts glycerol into 3-hydroxypropionaldehyde (3-HPA). The second reaction is the conversion of 3-HPA to 3-HP, catalyzed by an aldehyde dehydrogenase (Raj et al., 2008). This biosynthetic pathway is present in some natural producers of 3-HP, such as *Klebsiella pneumoniae* (Kumar et al., 2012; Ashok et al., 2013) and *Lactobacillus reuteri* (patent: US 20070148749 A1). The pathway has been heterologously expressed and studied in *Escherichia coli* and *Saccharomyces cerevisiae* (patent: WO 2011038364 A1; Chen et al., 2014; Jung et al., 2015; Kildegaard et al., 2015). Overexpression of the *E. coli* *aldH* gene in *K. pneumoniae* resulted in co-production of 1,3-propanediol (1,3-PDO) and 3-HP with a 3-HP titer of 24.4 g/L (Huang et al., 2012). In another study, 48.9 g/L of 3-HP was obtained in microaerobic fed-batch fermentation of *K. pneumoniae* WM3 expressing *aldH* from *E. coli* k12 (Huang et al., 2013). The recombinant *E. coli* BL21 expressing *dhaB* and *gdrAB* from *K. pneumoniae* and *KGSADH* from *Azospirillum brasilense* produced 38.7 g/L of 3-HP in fed-batch condition (Rathnasingh et al., 2009). In another report, deleting genes responsible for by products redirected the flux toward the 3-HP and resulted in higher titer of 57.3 g/L of 3-HP in fed-batch (Kim et al., 2014). Until now, the highest reported titer of 3-HP is 71.9 g/L using mutated aldehyde dehydrogenase (*ALDH*) (*GabD4*) from *Cupriavidus necator* in *E. coli* (Chu et al., 2015). Despite the noticeable progress in this area, microbial production of 3-HP has not yet reached industrial-scale productivity levels. Here we explored the possibility to use and alternative host for heterologous expression of the 3-HP synthetic pathway, the Gram-positive model organism *Bacillus subtilis*. It is non-pathogenic, generally recognized as safe (GRAS), suitable for large scale cultivation and has high growth rates. *B. subtilis* efficiently imports glycerol via a selective glycerol facilitator (GlpF; da Silva et al., 2009), and reaches a high growth rate on glycerol (μmax = 0.65 h^-1^; Kruyssen et al., 1980). When expressing the 3-HP synthetic pathway in a new host, the tolerance level toward 3-HP (Kildegaard et al., 2014) and the intermediate 3-HPA (Hao et al., 2008) can be a major consideration, resolved by a balanced expression of the two steps in the pathway (Sankaranarayanan et al., 2014). We expressed the 3-HP synthetic pathway from *K. pneumoniae* in *B. subtilis*. Since this particular glycerol dehydratase is sensitive to oxygen, micro-aerobic/anaerobic condition were required for optimal 3-HP production (Zhao et al., 2015). We carried out several rounds of strain and medium optimization, which resulted in a production strain capable of producing 10 g/L of 3-HP in shaking flasks, with the yield on glycerol ranging from 70 to 80%.

## Materials and Methods

### Strains and Plasmids

All the strains and plasmids used in this study are listed in **Table 1**. *B. subtilis* 168 trp^+^ was used as the host for genetic manipulations. Integrative plasmid pBS1C (BGSC, Columbus, USA) was used to clone *dhaB, gdrAB*, and *puuC* from *K. pneumoniae*. The codon-optimized construct containing the genes *dhaB123, gdrAB*, and *puuC* from *K. pneumoniae* was ordered as synthetic fragment (GenScript, Piscataway, NJ, USA) and cloned in the plasmid pBS1C. *E. coli* DH5α was used to amplify plasmids listed in **Table 1** and the *B. subtilis* 168 trp^+^ cells were transformed with the same plasmids using a previously described method (Fabret et al., 2002).

**Table 1:** Overview of the plasmids and strains used in this study.

Strain, plasmid	Description	Integrative site in *B. subtilis*	Source
**Strains**			
*B. subtilis* 168 trp^+^	Expression host and the source for *dhaS* gene		
*E. coli* DH5α	Cloning host		ATCC, USA
*K. pneumoniae* DSMZ 2026	Source for *dhaB123, gdrAB*, and *puuC* genes		DSM, Germany
**Plasmids**			
pBS1C-3	Codon-optimized synthetic pHyperspank promoter, *dhaB123, gdrAB, puuC* (*K. pneumoniae*) Amp, Cat	AmyE	This study
pMUTIN2-2	Deletion vector, *glpk* (*B. subtilis*), Amp, Erm		This study
pMAD	Deletion vector, *glpk* (*B. subtilis*), Amp, Erm		This study
**Recombinant strains**			
h-syn-KpDhaB-PuuC	*B. subtilis* 168 trp^+^ + pBS1C-3		This study
pBS1C-E	*B. subtilis* 168 trp^+^ + pBS1C empty		This study
pBS1C-E-ΔglpK	*B. subtilis* 168 trp^+^ + pBS1C empty and pMUTIN2-2		This study
h-syn-KpDhaB-PuuC-ΔglpK-i	*B. subtilis* 168 trp^+^ + pBS1C-3 and pMUTIN2-2		This study
h-syn-KpDhaB-PuuC-ΔglpK-ii	*B. subtilis* 168 trp^+^ + pBS1C-3 and pMAD		This study

### Genetic Manipulations

All synthetic genes (codon optimized) were under control of pHyperspank, a strong isopropyl β-D-1-thiogalactopyranoside (IPTG)-inducible promoter of *B. subtilis*. A strong ribosome binding site (RBS) from *B. subtilis* (ACATAAGGAGGAACTACT; kindly provided by Dominique Le Coq, INRA, France) was added before each synthetic fragment. The entire construct was ligated into the pBS1C integrative plasmid (BGSC, Columbus, USA) as an *Eco*RI–*Pst*I fragment. The integrative construct was used to transform *B. subtilis* 168 trp^+^. The Δ*glpk* strains were constructed either by using the single crossing over method described by Vagner et al. (1998) or a seamless and irreversible gene inactivation method (Arnaud et al., 2004). In the former, the pMUTIN2 plasmid carrying a portion of *glpK* is integrated into the *B. subtilis* genome through single cross-over event, resulting in disrupting the *glpK* gene. In the latter, the pMAD plasmid carrying a portion of the *glpK* gene is integrated at *glpK* locus in the *B. subtilis* genome in the first cross-over event, and the seamless and irreversible *glpK* knockout is obtained after the second single cross-over event. Stability of the recombinant constructs was tested by PCR using appropriate primers. All recombinant plasmids were cloned in *E. coli* DH5α and used to transform *B. subtilis* 168 trp^+^. All constructs were verified by restriction digestion and sequencing.

### Media and Growth

*Escherichia coli* strains and wild type (WT) *B. subtilis* were cultured in Luria–Bertani (LB) broth at 37°C in shaking flask (200 rpm) for genetic manipulations. For *E. coli* strains, ampicillin was added to the medium when needed at 100 μg/mL. Recombinant *B. subtilis* strains were grown in the minimal medium (M9; Harwood and Cutting, 1990) complemented with 12 g/L glycerol, 12 g/L glucose, and/or 1 g/L yeast extract, when required, at 37°C in shaking flask (200 rpm). Chloramphenicol 5 mg/mL and erythromycin 1 mg/mL were used when required to select *B. subtilis* transformants. Coenzyme B_12_ (15 μM) and IPTG (500 μM) were added in the medium. Recombinant *B. subtilis* strains were cultured in rich media used in shaking flask which was composed of 2 × M9: 40 g/L glycerol, 10 g/L yeast extract, and 3 g/L peptone. Seeds medium contained M9Y (M9 medium supplemented with 1 g/L yeast extract) with addition of 1% glucose. Cultivation medium contained M9Y and/or 2 × M9Y with addition of different concentration of glucose and glycerol (the initial concentration for glucose was 0.8% and for glycerol was about 2.7%). For batch cultivation in flask, a single colony was transferred into 50 mL falcon tube containing 10 mL seeds medium and cultured in shaker at 37°C and 200 rpm. Once the cell OD_600_ reached about 2, 1 mL of seeds culture was inoculated into 250 mL flask containing 35 mL cultivation medium. In fed-batch cultivation in flask, five glucose tablets (FeedBead Glucose discs) were added in each flask to release the glucose slowly in the medium. Flasks were shaken at 37°C and 200 rpm, and were kept in darkness. *B. subtilis* transformants were selected using chloramphenicol 5 mg/mL and erythromycin 1 mg/mL when required. Coenzyme B_12_ (15 μM) and IPTG (500 μM) were added in the medium. 3-HP production was followed in this setup for 2–4 days.

### Characterizing the Effect of 3-HP and 3-HPA on *B. subtilis* Growth

WT *B. subtilis* cells were grown in the M9 medium with 12 g/L glycerol as the sole carbon source. 3-HP was purchased from TCI Europe N.V. (Zwijndrecht, Belgium), and 3-HPA was chemically synthesized (Burgé et al., 2015a). 3-HP was tested in a range between 1 and 100 mM, while 3-HPA was tested between 0.02 and 20 mM. OD_600_ of the bacterial culture was continuously monitored for 24 h using a 96-well plate reader (Biotek, USA), at 37°C, with three biological replicates.

### Proteomics Analysis

Cells were collected at four different time points during fermentation: 0, 24, 48, and 72 h. Cell pellets were re-suspended in an sodium dodecyl sulfate (SDS) lysis buffer containing 4% SDS in 100 mM triethylammonium bicarbonate pH 8.0, 10 mM ethylenediaminetetraacetic acid and a protease inhibitor cocktail (Roche). The cell extract was boiled at 90°C for 10 min followed by sonication for 30 s. The cell debris was removed by centrifugation. The protein pellet obtained after chloroform/methanol precipitation was dissolved in denaturation buffer containing 8 M urea in 10 mM Tris–HCl pH 8.0. Protein concentration was measured by Bradford protein assay. A total of 100 μg of each sample were separated on a Mini-Protean^®^ TGX^TM^ 4–20% gradient gel (Bio-Rad) and stained with Bio-Safe^TM^ Coomassie (Bio-Rad). Regions corresponding to the size of “DhaB1” were cut and in-gel trypsin (Pierce^TM^) digestion was performed as described (Shevchenko et al., 2007). Peptides were desalted using C-18 stage-tips and analyzed on the Orbitrap Fusion^TM^ Tribrid^TM^ (Thermo Fischer Scientific). An inclusion list consisting of *m/z* values in the range of 400–1600 Da and charge states +2 and +3 only was incorporated into the instrument method. Acquired mass spectra were processed with the MaxQuant software suite (v.1.5.1.0; Cox et al., 2009).

### *In silico* Simulation of the Metabolism

The genome-scale metabolic model iYO844 was obtained in a COBRA Toolbox (Schellenberger et al., 2011) compatible SBML format from http://systemsbiology.ucsd.edu. Two reactions (ALCD19_D and ALCD19_L) involving in alcohol dehydrogenases were removed from the model due to the determined roles of YhdN in methylglyoxal resistance (Chandrangsu et al., 2014). Although YhcW and YvoE had been identified as glycerol-3-phosphatase (Lindner et al., 2012), we did not include corresponding reaction into the model due to the low *in vivo* activity of these enzymes (data not shown). The relevant reactions for 3-HP production (Supplementary Figure S5) were added to the model. The 3-HP production envelopes were generated using COBRA toolbox in Matlab (MathWorks, Inc., Natick, MA, USA). For the simulation of aerobic growth in minimal medium M9, the carbon uptake (glycerol and/or glucose) had a limit of -10 mmol gDW^-1^ h^-1^ as the maximum uptake rate. Aerobic conditions were simulated by setting the lower and upper limits for the O_2_ exchange flux to free (-1000 to 1000 mmol gDW^-1^ h^-1^). In addition, the uptake rates of sulfate and ammonium were set to free, as were the K^+^, Na^+^, Mg^2+^, Ca^2+^, Fe^3+^, CO_2_, H_2_O, and H^+^ uptakes. For reaction knockout simulations, the minimum and maximum fluxes of the corresponding reactions were set to 0. The reactions available to knockout was reduced to a pool of approximately 400 reactions that excluded exchange reactions, transporter reactions, and essential reactions.

### Metabolite Analyses

Glycerol, 3-HP, and lactate concentrations were measured using high performance liquid chromatography (HPLC) (Ultimate 3000, Dionex). 1 mL of culture samples were centrifuged at 12,000 × *g* for 10 min, then 200 μL of 8 mM H_2_SO_4_ was added to 800 μL of the supernatant and filtered through a 0.20 μm pore size nitrocellulose filter (Sartorius Stedim). Separation was performed on a Bio-Rad Aminex HPX-87H column (300 mm × 7.8 mm; Bio-Rad, Richmond, USA) connected to a VWD-3100 detector (Thermo Scientific Dionex) with 0.5 or 8 mM H_2_SO_4_ as the eluent, as described by Burgé et al. (2015b). All samples were analyzed in three replicates. Gas chromatography mass spectrometry (GC-MS) was also used to quantify the 3-HP and lactate. All samples were derivatized with methyl chloroformate. For quality control, a mixed pooled sample (QC sample) was created by taking an aliquot from each sample. Testing of matrix effects was performed by spiking/dilution of QC samples. The GC-MS data were processed by PARAFAC2 model from MS-Omics^1^.

## Results and Discussion

### Sensitivity of *B. subtilis* to 3-HPA and 3-HP

**FIGURE 1:**
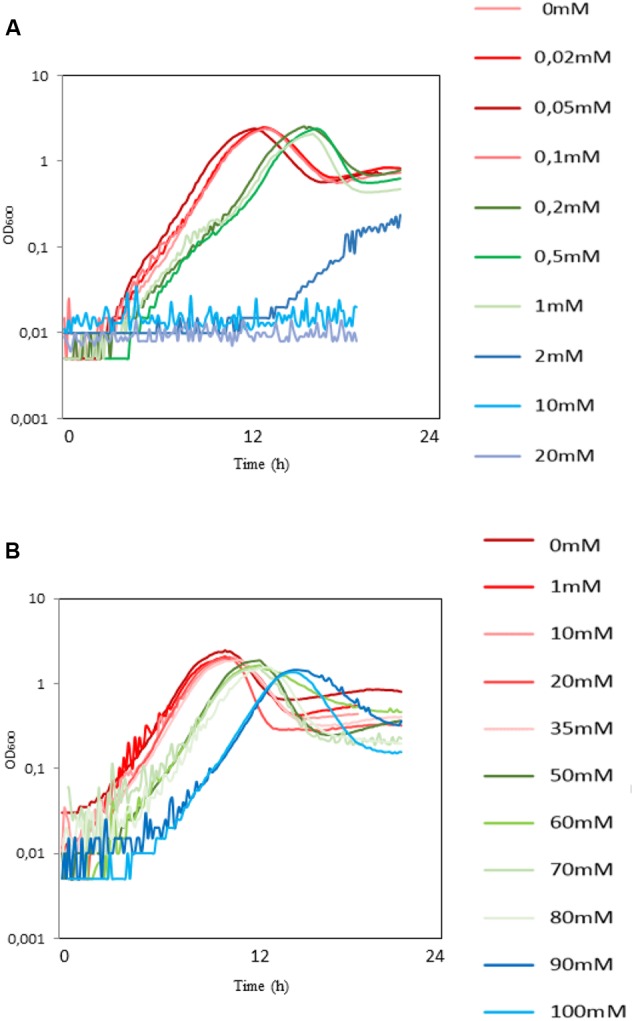
**The toxic effect of 3-HPA and 3-HP on the growth of wild type (WT) *B. subtilis.*** Growth curves of the WT *B. subtilis* growing for 24 h on the M9 minimal medium with glycerol as carbon source supplemented with **(A)** 3-HPA (0.02–20 mM) and **(B)** 3-HP (1–100 mM). Both growth rate and the duration of lag phase was affected as the concentration of either 3-HPA or 3-HP increased which are classified in three groups of red, green, and blue colors representing the effect of either of the compounds on growth of WT *B. subtilis* from low to high, respectively. Respective concentrations of 3-HP and 3-HPA are indicated next to each curve.

The product of the first step of conversion of glycerol to 3-HP is 3-HPA, known to be toxic to some bacteria (Hao et al., 2008). We examined the effect of 3-HPA and 3-HP on *B. subtilis* growing on the M9 minimum medium supplemented with glycerol. The 3-HPA exhibited a strong inhibitory effect from very low concentrations. The specific growth rate in the 0–0.1 mM range (red-colored group, **Figure 1A**) was 0.55 h^-1^. It decreased to 0.27 h^-1^ for the 0.2–1 mM range (green-colored group, **Figure 1A**), and the growth was completely abolished in the 2–20 mM range (blue-colored group, **Figure 1A**). The lag phase was also prolonged as the concentration of 3-HPA increased (9:30 h, 14:30 h, and 20 h for the three groups, respectively). *B. subtilis* cells were much more tolerant toward 3-HP. The specific growth rate in the 0–35 mM range (red-colored group, **Figure 1B**) was 0.59 h^-1^. It decreased slightly to 0.56 h^-1^ for the 50–80 mM range (green-colored group, **Figure 1B**) and further to μ = 0.54 h^-1^ for the 90–100 mM range (blue-colored group, **Figure 1B**). The lag phase was also affected as the concentration of 3-HP increased (7:30 h, 9:30 h, and 13:30 h for the three groups, respectively). It is important to note that the 3-HP sample was buffered, so the growth effects were not due to the acidification of the medium. Regarding the tolerance level of other microorganisms toward 3-HP, is has been reported that *E. coli* has normal growth in M9 with addition of 100 mM of 3-HP, while the growth significantly decreases with 3-HP concentrations in the range of 330–440 mM (Chun et al., 2014). There has also been a report on *S. cerevisiae* strains tolerant to 50 g/L 3-HP obtained through adaptive laboratory evolution (Kildegaard et al., 2014). Our results indicate that *B. subtilis* is fairly tolerant to 3-HP, but highlight the importance of preventing the accumulation of the 3-HPA in the cell.

### Expression of the Codon-optimized 3-HP Pathway from *K. pneumoniae* Leads to the Production of 3-HP

**FIGURE 2:**
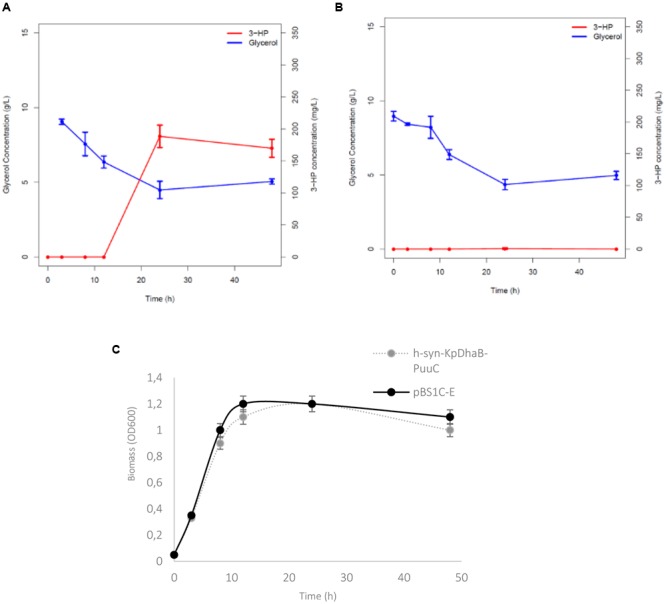
**Time course of glycerol consumption and 3-HP production. (A)** Recombinant strain overexpressing codon-optimized glycerol dehydratase and its activators (*dhaB123, gdrAB*) and the aldehyde dehydrogenase (*PuuC*) from *K. pneumoniae* under control of pHyperspank promoter (h-*syn-KpDhaB-PuuC*) and **(B)** the control strain with the empty plasmid (pBS1C-E), cultivated in M9 medium with 12 g/L glycerol, induced by IPTG and B_12_. **(C)** Growth profiles of h-*syn-KpDhaB-PuuC* and pBS1C-E strains. Glycerol consumption is shown in blue, and 3-HP production in red. Average values from three independent biological replicates are shown, with the standard deviation.

**FIGURE 3:**
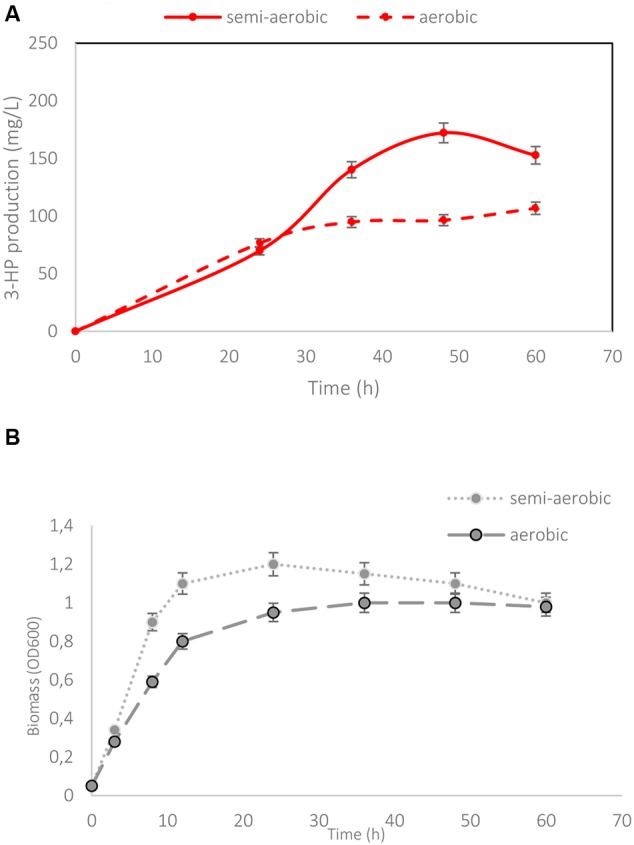
**Effect of the aerobic and semi-aerobic condition on 3-HP production. (A)** Cultivation of the strain h-*syn-KpDhaB-PuuC* in aerobic condition leads to decreased 3-HP production compared to semi-aerobic condition. **(B)** Growth profile of h-*syn-KpDhaB-PuuC* in aerobic and semi-aerobic condition. 3-HP production is represented in solid and dotted lines for semi-aerobic and aerobic conditions, respectively. Average values from two independent biological replicates are shown, with the standard deviation.

The *dhaB* and *puuC*, coding for the B_12_-dependent glycerol dehydratase and aldehyde dehydrogenase from *K. pneumoniae*, were codon optimized for *B. subtilis* (GenScript). The optimization concerned 23% of the codons. All modified codons can be seen in an alignment of non-optimized and optimized sequences provided in Supplementary Figure S1. The synthetic genes were expressed in *B. subtilis* from a single construct in pBS1C-3, resulting in the strain h-syn-*KpDhaB*-*PuuC*. The entire construct was under the control of a strong promoter pHyperspank (Vagner et al., 1998), and each gene was preceded by the strong RBS (ACATAAGGAGGAACTACT). The *B. subtilis* strain containing the empty pBS1C plasmid (*pBS1C-E* strain) was used as the control. We confirmed the expression of the glycerol dehydratase and aldehyde dehydrogenase in the h-syn-*KpDhaB*-*PuuC* crude extract by mass spectrometry (MS/MS spectra shown in Supplementary Figure S2), which were detected with excellent coverage and no ambiguity. The h-syn-*KpDhaB*-*PuuC* and *pBS1C-E* strains were cultured and samples were collected at regular intervals after induction to measure the growth rate, glycerol consumption and 3-HP production (**Figure 2**). The growth rates (Supplementary Figure S3) and glycerol consumption (**Figure 2**) were comparable for both strains. After 24 h, 3-HP was detected in the h-syn-*KpDhaB*-*PuuC* culture at 180 mg/L (**Figure 2A**). As expected, no 3-HP was detected in the control strain *pBS1C-E* (**Figure 2B**). Growth profiles of h-syn-*KpDhaB*-*PuuC* and *pBS1C-E* are shown in **Figure 2C**. Quantification of 3-HP was confirmed by an independent method using GC-MS (Supplementary Figure S4). Since the growth rate was not affected by the expression of the heterologous pathway, we concluded that 3-HPA is not accumulating in the production strain, and no further balancing of the expression of the two reaction steps was needed. The B_12_-dependent DhaB from *K. pneumoniae* is sensitive to excessive oxygen (Zhao et al., 2015), so we compared the production of 3-HP in micro-aerobic and fully aerobic conditions (**Figure 3A**) and their respective growth profile (**Figure 3B**). In the semi-aerobic condition the h-syn-*KpDhaB*-*PuuC* clearly performed better, so this condition was used throughout the following optimization steps. These results altogether indicate that the codon-optimized synthetic version of the 3-HP biosynthetic pathway was correctly expressed and functional in *B. subtilis*. There has been a report on positive effect of glucose addition to the 3-HP producing strain of *E. coli* grown on glycerol, increasing the 3-HP production by reducing the imbalance between the first and second step of the pathway (Niu et al., 2016). To test whether this effect applies to our strain, we grew the h-syn-*KpDhaB*-*PuuC* in two conditions: (1) 1.2% glycerol as the sole carbon source and (2) 1.2% glycerol supplemented with 1.2% glucose. Our results showed no significant increase in 3-HP production when adding glucose to the culture. The 3-HP production was identical in both conditions (approximately 200 mg/L with less than 3% difference). We concluded that this effect does not apply to our production strain.

### *In silico* Identification of Targets for Increased Production of 3-HP

**FIGURE 4:**
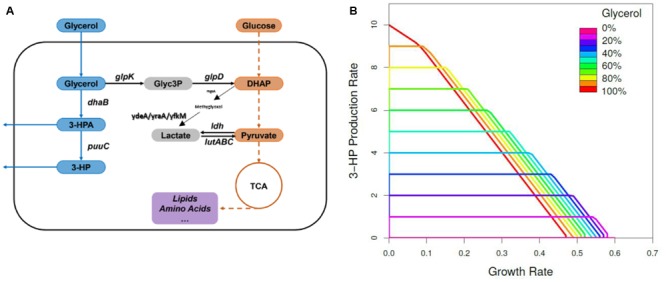
**The metabolic pathways involved in 3-HP biosynthesis and prediction of maximum 3-HP production rate. (A)** Biosynthesis of 3-HP was achieved by integration of a heterologous metabolic route into *B. subtilis*. The metabolite abbreviations are as follows: Glyc3P, glycerol 3-phosphate; DHAP, dihydroxyacetone phosphate. The gene names are as follows: *glpK*, glycerol kinase; *glpD*, glycerol-3-phosphate dehydrogenase; *dhaB*, glycerol dehydratase; *dhaS*, aldehyde dehydrogenase; *ldh*, L-lactate dehydrogenase; *lutABC*, lactate catabolic; *mgsA*, methylglyoxal synthase. **(B)** Predicted maximum 3-HP production rate limits as a function of glycerol proportion in the total carbon source, in the glucose- and glycerol-fed cultures, based on the iYO844 GEM. The color scale denotes the ratio of glycerol in the carbon source from 0 (velvet) to 100 (red).

The genome-scale metabolic model of *B. subtilis* (iYO844; Oh et al., 2007), with the addition of the heterologous 3-HP synthetic pathway (Supplementary Figure S5 and **Figure 4A**) was used to predict targets for optimizing the 3-HP production. As shown in **Figure 4B**, the predicted maximum growth rate was achieved when using glucose as the sole carbon source, while the predicted maximum 3-HP production rate was reached when using glycerol as the sole carbon source. When growing on both glucose and glycerol, the flux of glycerol to 3-HP biosynthesis is negatively related to the flux of glycerol to biomass, indicating that the reduction of glycerol conversion to biomass should improve the 3-HP yield. Our *in silico* analysis (Supplementary Table S1) was performed to identify single gene knockouts which are expected to improve 3-HP production under these conditions. As expected from the literature (Jung et al., 2014; Tsuruno et al., 2015), the deletion of the gene *glpK*, which encodes glycerol kinase, was the top candidate for redirecting the metabolic flux toward 3-HP biosynthesis when grown on a combination of glucose and glycerol. The *glpK* strain was the only knockout predicted to achieve high productivity, while maintaining an acceptable growth rate.

### Inactivation of *glpK* Leads to Increased 3-HP Production

**FIGURE 5:**
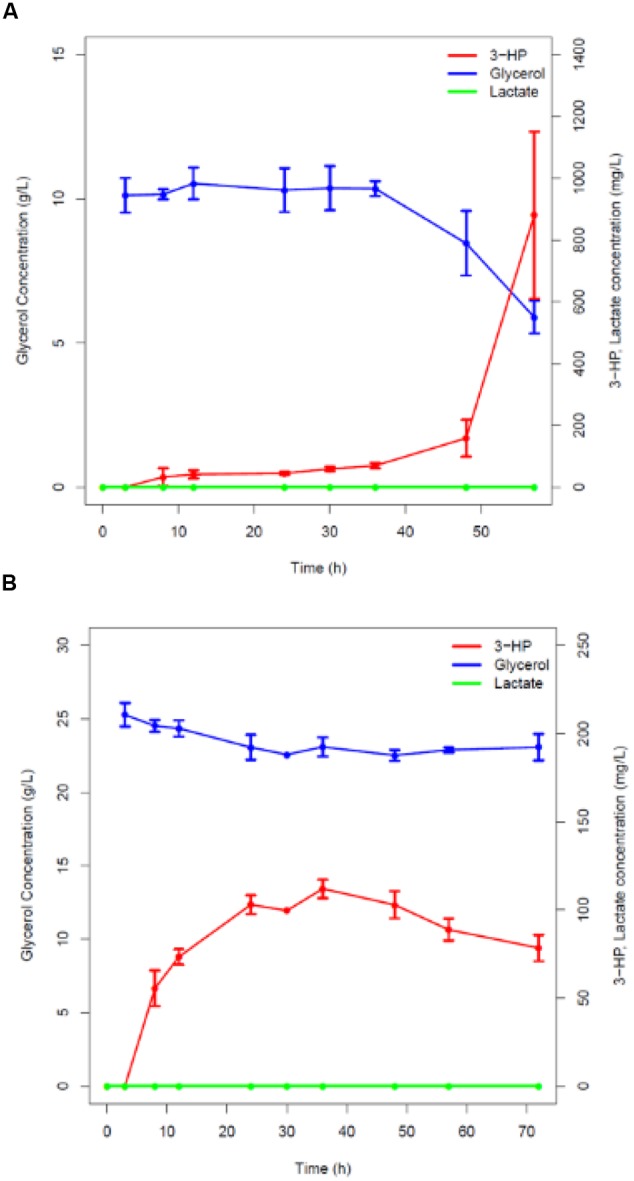
**Time course of glycerol consumption and lactate and 3-HP production. (A)** Recombinant strain with a knocked-out glycerol kinase (*glpk*) overexpressing codon-optimized glycerol dehydratase and its activators (*dhaB123, gdrAB*) and the aldehyde dehydrogenase (*puuC*) from *K. pneumoniae* under control of pHyperspank promoter (strain h-*syn-KpDhaB-PuuC*-Δ*glpK-i*) cultivated in the M9 medium with glucose, induced by IPTG, and supplemented with B_12_ and **(A)** 12 g/L glycerol, **(B)** 25 g/L glycerol. Glycerol consumption is shown in blue, lactate production in green and 3-HP production in red. Average values from three independent biological replicates are shown, with the standard deviation.

GlpK catalyzes the formation of glycerol-3-phosphate from glycerol, which is further converted to dihydroxyacetone phosphate to enter glycolysis (Holmberg et al., 1990). Based on the *in silico* prediction presented above, the *glpK* gene was knocked out using the method reported by Vagner et al. (1998) resulting in strains h-syn*-KpDhaB-PuuC-*Δ*glpK-i* and *pBS1C-E-*Δ*glpK*, respectively. This deletion naturally suppressed the growth on glycerol as the sole carbon source (data not shown). We therefore cultured the strains h-syn*-KpDhaB-PuuC-*Δ*glpK-i* and *pBS1C-E-*Δ*glpK* in the M9 minimum medium supplemented with glucose up to the induction point at OD_600_ = 1.2. 3-HP was detected in the h-syn*-KpDhaB-PuuC-*Δ*glpK-i* culture 8 h after induction (50 mg/L) and its concentration increased to the maximum of 1 g/L after 60 h (**Figure 5A**). As expected, non-induced and *pBS1C-E-Δglpk* produced no 3-HP (Supplementary Figure S6). The glycerol uptake was not reduced in h-syn*-KpDhaB-PuuC-*Δ*glpK-i* (**Figure 5A**) compared to the control strain (Supplementary Figure S6A), indicating that the *glpK* knockout successfully redirected the flux toward 3-HP production. Next, we increased the glycerol concentration to 25 g/L, but this failed to increase the 3-HP titer (**Figure 5B**). This indicated that one (or more) of the pathway components does not operate at the maximum rate.

### 3-HP Production Increased to 10 g/L in the Semi-defined and Rich Media

**FIGURE 6:**
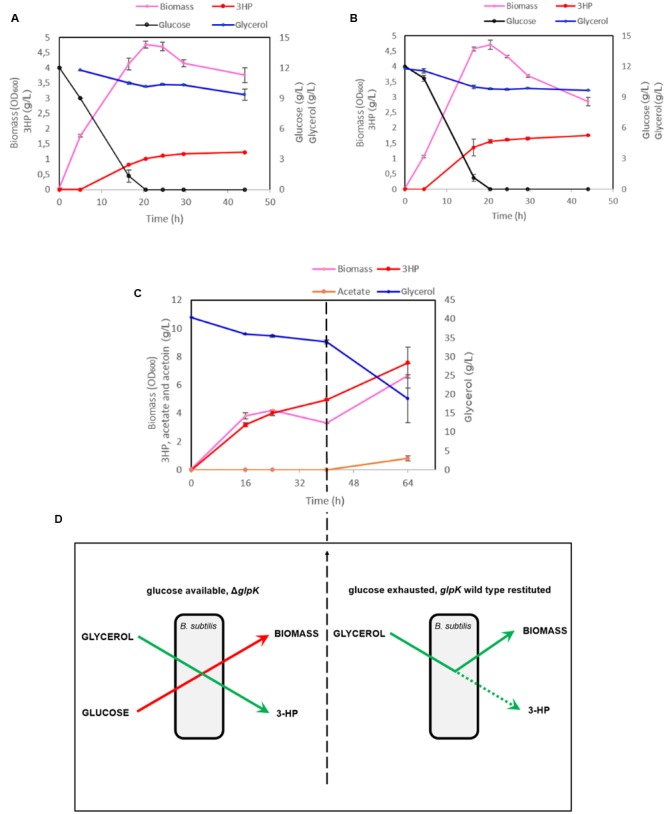
**Cultivation of the strain h-*syn-KpDhaB-PuuC*-Δ*glpK-i* in the semi-defined and rich medium leads to increased 3-HP production.** IPTG, glycerol and coenzyme B_12_ were added to the medium at 5 h **(A)** and at the beginning of cultivation **(B,C)**. Glycerol and glucose consumption are shown in blue and black, respectively, 3-HP and acetate production are represented in red and orange, respectively, and biomass is shown in pink. Average values from three independent biological replicates are shown, with the standard deviation. **(D)** Schematic representation for explaining the loss of *ΔglpK* mutation after prolonged incubation of the production strain in the medium with glucose and glycerol. The restituted wild type (WT) *glpK* diverts the flux from glycerol to biomass, and reduces the flux to 3-HP, thus decreasing the 3-HP yield on glycerol. In the early stage of the experiment (left), glucose is used for biomass production, and a high yield of 3-HP on glycerol is achieved. At the late stage of the experiment (right), glucose is exhausted, *glpK* WT is restituted, and 3-HP yield on glycerol is diminished.

**FIGURE 7:**
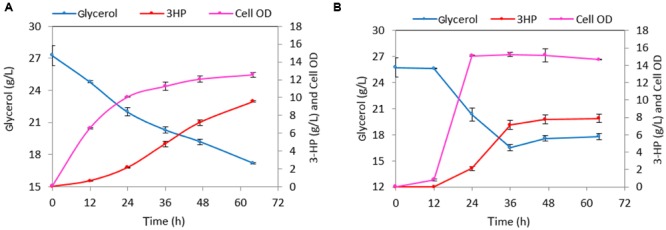
**Cultivation of the strain h-*syn-KpDhaB-PuuC*-Δ*glpK-ii* in 2 × M9Y medium and rich medium for 3-HP production. (A)** h-*syn-KpDhaB-PuuC*-Δ*glpK-ii* in 2 × M9Y medium, **(B)** h-*syn-KpDhaB-PuuC*-Δ*glpK-ii* in rich medium h-*syn-KpDhaB-PuuC*-Δ*glpK-i* in 2 × M9Y medium. Glycerol consumption is shown in blue, 3-HP production is represented in red, and biomass is shown in pink. Average values from two independent biological replicates are shown, with the standard deviation.

The maximal OD_600_ reached by the strain h-syn*-KpDhaB-PuuC-*Δ*glpK-i* in the M9 medium was 3.8. To reach higher cell density, we further cultured the production strain in M9 supplemented with 1 g/L yeast extract. The maximal cell OD_600_ reached 4.7 (**Figure 6A**) after 20.5 h, which is about 24% higher than in the M9 medium. This strain produced 1.23 g/L of 3-HP and consumed 2.4 g/L glycerol at the end of the cultivation (after 44 h), with a yield of 0.51 g 3-HP/g glycerol. The less than 100% yield is common in other bacterial cell factories, and is usually due to competing pathways, such as 1,3-PDO production (Dishisha et al., 2015), or conversion of glycerol to lactate via the methylglyoxal pathway (Landmann et al., 2011). We also observed that glucose and glycerol were consumed simultaneously from 5 to 20.5 h, which suggested that the expression of *glpF*, the gene encoding glycerol transporter, was most probably not negatively regulated by the presence of glucose. Since there is only one copy of *dhaB123, gdrAB*, and *puuC* genes in h-syn*-KpDhaB-PuuC-*Δ*glpK-i* strain, we inferred that the expression of these genes brings no obvious metabolic burden. Therefore, we induced the expression of synthetic pathway at the beginning of the cultivation. As shown in the **Figure 6B**, the early induction had no obvious negative effect on cell growth. A titer of 1.56 and 1.76 g/L 3-HP was accumulated at 20.5 and 44 h, respectively, and the 3-HP yield on glycerol reached 0.83 g/g. The ability of h-syn*-KpDhaB-PuuC-*Δ*glpK-i* to produce 3-HP was also evaluated in a rich medium. As shown in **Figure 6C**, a final 3-HP titer of 7.6 g/L was achieved and 21.4 g/L glycerol was consumed after a 64 h cultivation. However, the average 3-HP yield was only 0.35 g/g glycerol, much lower than the one obtained in the semi-defined medium. The glycerol consumption rate significantly increased from 40 to 64 h, accompanied by an increase in growth rate and biomass accumulation. In the same period, the 3-HP yield decreased from 0.78 g/g (0–40 h) to 0.17 g/g (40–64 h) glycerol. We suspected that this growth transition and the decrease in yield was caused by the instability of the Δ*glpK* mutation. Under our cultivation conditions, it is possible for the pMUTIN2-2 to exit the chromosome, resulting in the restitution of the functional *glpK*. Before the glucose in the medium is exhausted, up to 40 h, glucose is converted into biomass and glycerol is efficiently converted to 3-HP (80% yield; **Figure 6D**). Once the glucose is exhausted, selection pressure restitutes the WT *glpK*, and glycerol is then mainly converted to biomass, with the 3-HP yield dropping to 40% (**Figure 6D**). To test this assumption, we plated the cultures grown in the production setup after 64 h, and counted the colony forming units (CFU) on M9 supplemented with either glucose or glycerol (10 g/L). The CFU counts of the strain h-syn*-KpDhaB-PuuC-*Δ*glpK-i* on M9-glycerol plate was 71 ± 11% of that on M9-glucose plates, indicating that a significant sub-population of cells have restituted WT *glpK*. This explains the reduced yield of 3-HP on glycerol after 40 h of cultivation, since the loss of the Δ*glpK* mutation diverts the flux from glycerol to biomass, and reduces the flux to 3-HP (**Figure 6D**). The loss of the *glpK* knockout during cultivation could also be detected by PCR, checked at four different time points (Supplementary Figure S7A). To counter this effect, we constructed an irreversible Δ*glpK* strain using the method described by Arnaud et al. (2004). Using this method, the pMAD plasmid is first integrated at the *glpK* locus in the *B. subtilis* genome, and then the deletion is achieved when the plasmid leaves the chromosome in the second crossing over event. The resulting strain was named h-syn*-KpDhaB-PuuC-*Δ*glpK-ii*. Stability of the *glpK* knockout in this strain was confirmed by PCR at four different time points throughout the cultivation (Supplementary Figure S7B). Moreover, full stability of the integrated heterologous pathway was also confirmed by PCR at the same time points (Supplementary Figure S7C). We also checked the expression of the heterologously expressed proteins in crude extracts by using mass spectrometry proteomics, and have consistently detected both the DhaB and PuuC at all time points throughout the cultivation (Supplementary Figure S2). Compared to h-syn*-KpDhaB-PuuC-*Δ*glpK-i*, the h-syn*-KpDhaB-PuuC-*Δ*glpK-ii* was superior in terms of 3-HP titer (10 g/L vs 7.6 g/L) (**Figures 7A,B**) and average yield on glycerol (0.79 g/g vs 0.35 g/g), both of which remained stable throughout the experiment. This confirmed the hypothesis of a positive selective pressure on restoring the *glpK*, and highlighted the importance of using a stable knockout.

## Conclusion and Perspectives

*Bacillus subtilis* has the potential to be considered as a microbial host for production of 3-HP due to several reasons including its efficient glycerol import system (da Silva et al., 2009) and high growth rate on glycerol (μmax = 0.65 h^-1^; Kruyssen et al., 1980) which compared to *E. coli* (μmax = 0.26 h^-1^; Chaudhary et al., 2012) is an advantage. It can reach high optical density (OD) in fermentation (Park et al., 1992) and has the ability to grow on simple media (Demain, 1958). *B. subtilis* can efficiently grow at high temperatures which reduces cooling costs during fermentation (Fu et al., 2016). In addition, there are number of developing novel and useful tools for genetic modification of *B. subtilis* (Dong and Zhang, 2014; Westbrook et al., 2016). Therefore, here we demonstrated that *B. subtilis* is a viable host for conversion of glycerol to 3-HP, with the production strain h-syn*-KpDhaB-PuuC-*Δ*glpK-ii* reaching a titer of 10 g/L in shake flasks. This is already comparable to the best benchmark strains of *E. coli* in shake flasks (Rathnasingh et al., 2009; Chu et al., 2015). Further optimization steps should include increasing the uptake rate for glycerol, by upregulating the expression of *glpF*, and export of 3-HP, which presumably takes place via the lactate transporter (Maris et al., 2004). More active dehydrogenases such as mutated *ALDH* (GabD4; Chu et al., 2015) could be used for further production improvement. Transferring the B_12_ production gene cluster to *B. subtilis* from its close relative *Bacillus megaterium* would significantly reduce the cost of production and should be considered. Adaptive laboratory evolution aimed at achieving higher glycerol uptake and utilization of chemostat conditions to neutralize the acidification from produced 3-HP, or on-line extractors to continuously remove 3-HP, should be reasonably expected to result in a robust and exploitable production strain.

## Author Contributions

AK, TC, VR, and CS-B performed the experiments. BJ, IS, and AG performed the computational analyses. AK, TC, BJ, AG, DF, and IM analyzed the data. AK and IM wrote the manuscript.

## Conflict of Interest Statement

The authors declare that the research was conducted in the absence of any commercial or financial relationships that could be construed as a potential conflict of interest.

## Footnotes

1 http://msomics.com/
