# Supplementary material for: Conversion of Glycerol to 3-Hydroxypropanoic Acid by Genetically Engineered Bacillus subtilis
Source: Front Microbiol. 2017 Apr 18;8:638. doi: 10.3389/fmicb.2017.00638 (PMC5394112; doi:10.3389/fmicb.2017.00638)
Supplement: Supplementary file 1 [file Data_Sheet_1.PDF]

Conversion of glycerol to 3-hydroxypropanoic acid by genetically engineered *Bacillus subtilis*

Aida Kalantari[1,2], Tao Chen[1,7], Boyang Ji[1], , Ivan Andreas Stancik[1,4], Vaishnavi Ravikumar[1], Damjan Franjevic[4], Claire Saulou-Bérion[3], Anne Goelzer[5] and Ivan Mijakovic[1,6]*

[1]Systems and Synthetic Biology Division, Department of Biology and Biological Engineering, Chalmers University of Technology, Sweden

[2]Chaire Agro-Biotechnologies Industrielles, AgroParisTech, Reims, France

[3]UMR GMPA, AgroParisTech, INRA, Université Paris-Saclay, Thiverval Grignon, France

[4]Department of Biology, Faculty of Science, University of Zagreb, Zagreb, Croatia

[5]MaIAGE, INRA, Université Paris-Saclay, Jouy-en-Josas, France

[6]Novo Nordisk Foundation Center for Biosustainability, Technical University of Denmark, Lyngby, Denmark

[7]Key Laboratory of Systems Bioengineering (Ministry of Education), Tianjin University, Tianjin, China

*Correspondence: Ivan Mijakovic, ivan.mijakovic@chalmers.se

Keywords: 3-hydroxypropanoic acid, glycerol, *Bacillus subtilis*, metabolic engineering, glycerol kinase knock-out

Supplementary material

Table S1. The predicted maximum growth rate and maximum 3-HP production rate in *B. Subtilis* wide type and single gene deletion mutants.

Reactions	Enzymes	Reaction Formulas	Subsystem	EC	Genes	Glucose			Glycerol			Glucose + Glycerol		
						G_{max}	P_{maxG}	$P_{maxG/2}$	G_{max}	P_{maxG}	$P_{maxG/2}$	G_{max}	P_{maxG}	$P_{maxG/2}$
WT						***0.6007***	***0.0000***	***0.0003***	***0.4750***	***0.0000***	***5.4681***	***0.5401***	***0.0000***	***5.0002***
ACONT	aconitase	[c] : cit <==> icit	Carbohydrates and related molecules	4.2.1.3	BG10478	0.5721	0.0000	0.0003	***0.4185***	0.0000	5.5946	***0.5037***	***0.0000***	5.0002
AKGD	2-oxoglutarate dehydrogenase	[c] : akg + coa + nad --> co2 + nadh + succoa	Carbohydrates and related molecules	1.2.4.2	BG10210, BG10273, BG10272	***0.5531***	0.0000	0.0003	0.4603	0.0000	5.5116	***0.5090***	***0.0000***	5.0001
ATPS4r	ATP synthase (four protons for one ATP)	adp[c] + (4) h[e] + pi[c] <==> atp[c] + (3) h[c] + h2o[c]	Membrane bioenergetics	3.6.3.14	BG10816+ BG10822+ BG10819+ BG10817+ BG10820+ BG10815+ BG10814+ BG10818+ BG10821	***0.0792***	0.0000	***0.0000***	***0.0778***	0.0000	***2.7500***	***0.0792***	***0.0000***	***2.7500***

G_{max}: predicted maximum growth rate, P_{maxG}: predicted maximum 3-HP production rate at maximum growth rate, $P_{maxG/2}$: predicted maximum 3-HP production rate at half of the maximum growth rate, “-”: the deletion of corresponding reaction lead to no growth.

Reactions	Enzymes	Reaction Formulas	Subsystem	EC	Genes	Glucose			Glycerol			Glucose + Glycerol		
						G_{max}	P_{maxG}	$P_{maxG/2}$	G_{max}	P_{maxG}	$P_{maxG/2}$	G_{max}	P_{maxG}	$P_{maxG/2}$
WT						***0.6007***	***0.0000***	***0.0003***	***0.4750***	***0.0000***	***5.4681***	***0.5401***	***0.0000***	***5.0002***
CS	citrate synthase	[c] : accoa + h2o + oaa --> cit + coa + h	Carbohydrates and related molecules	4.1.3.7	BG10854, BG10855	0.5721	0.0000	0.0003	***0.4185***	0.0000	5.5946	***0.5037***	0.0000	5.0002
CYOO3	cytochrome-c oxidase (H+/e- = 2)	(2) focytc[c] + (6) h[c] + (0.5) o2[c] --> (2) ficytc[c] + (4) h[e] + h2o[c]	Membrane bioenergetics	1.9.3.1,	BG10218+ BG10217+ BG10216+ BG10215	***0.4436***	0.0000	***0.0002***	***0.3563***	0.0000	5.2414	***0.4021***	***0.0000***	5.0001
CYOR3m	cytochrome-c reductase (menaquinol 7: 3 protons)	(2) ficytc[c] + h[c] + mql7[c] --> (2) focytc[c] + (3) h[e] + mqn7[c]	Membrane bioenergetics	1.10.2.2,	BG11327+ BG11326+ BG11325	***0.4436***	0.0000	***0.0002***	***0.3563***	0.0000	5.2414	***0.4021***	***0.0000***	5.0001
FUM	fumarase	[c] : fum + h2o <==> mal-L	Carbohydrates and related molecules	4.2.1.2	BG10384	-	-	-	***0.4255***	0.0000	5.5946	***0.3218***	***0.0000***	***3.2793***

G_{max}: predicted maximum growth rate, P_{maxG}: predicted maximum 3-HP production rate at maximum growth rate, $P_{maxG/2}$: predicted maximum 3-HP production rate at half of the maximum growth rate, “-”: the deletion of corresponding reaction lead to no growth.

Reactions	Enzymes	Reaction Formulas	Subsystem	EC	Genes	Glucose			Glycerol			Glucose + Glycerol		
						G_{max}	P_{maxG}	$P_{maxG/2}$	G_{max}	P_{maxG}	$P_{maxG/2}$	G_{max}	P_{maxG}	$P_{maxG/2}$
WT						***0.6007***	***0.0000***	***0.0003***	***0.4750***	***0.0000***	***5.4681***	***0.5401***	***0.0000***	***5.0002***
GAPD_NAD	glyceraldehyde-3-phosphate dehydrogenase (phosphorylating)	[c] : g3p + nad + pi --> 13dpg + h + nadh	Carbohydrates and related molecules	1.2.1.12	BG10827	0.6007	0.0000	0.0003	***0.2745***	0.0000	5.5946	***0.5072***	***0.0000***	5.0002
GLYK	glycerol kinase	[c] : atp + glyc --> adp + glyc3p + h	Carbohydrates and related molecules	2.7.1.30	BG10187	0.6007	***0.0003***	0.0003	-	-	-	***0.3209***	***5.0002***	5.0002
PC	pyruvate carboxylase	[c] : atp + hco3 + pyr --> adp + h + oaa + pi	Carbohydrates and related molecules	EC-6.4.1.1	BG12660	0.6007	0.0000	0.0003	***0.4308***	0.0000	5.4681	0.5401	0.0000	5.0002
PGK	phosphoglycerate kinase	[c] : 13dpg + adp <==> 3pg + atp	Carbohydrates and related molecules	2.7.2.3	BG11062	-	-	-	***0.2745***	0.0000	5.5946	***0.5072***	***0.0000***	***4.2679***
PYK	pyruvate kinase	[c] : adp + h + pep --> atp + pyr	Carbohydrates and related molecules	EC-2.7.1.40	BG12661	0.6007	0.0000	0.0003	***0.4457***	0.0000	5.5116	0.5306	0.0000	5.0002
RPI	ribose-5-phosphate isomerase	[c] : r5p <==> ru5p-D	Carbohydrates and related molecules	5.3.1.6	BG10942	0.5462	0.0000	0.0003	***0.4322***	0.0000	5.4535	***0.4911***	***0.0000***	5.0001
SUCD1	succinate dehydrogenase	[c] : fad + succ <==> fadh2 + fum	Carbohydrates and related molecules	1.3.99.1	BG10353+ BG10352+ BG10351	-	-	-	***0.4353***	0.0000	5.5946	***0.3039***	***0.0000***	***3.4414***

G_{max}: predicted maximum growth rate, P_{maxG}: predicted maximum 3-HP production rate at maximum growth rate, $P_{maxG/2}$: predicted maximum 3-HP production rate at half of the maximum growth rate, "-": the deletion of corresponding reaction lead to no growth.

Reactions	Enzymes	Reaction Formulas	Subsystem	EC	Genes	Glucose			Glycerol			Glucose + Glycerol		
						G_{max}	P_{maxG}	$P_{maxG/2}$	G_{max}	P_{maxG}	$P_{maxG/2}$	G_{max}	P_{maxG}	$P_{maxG/2}$
WT						***0.6007***	***0.0000***	***0.0003***	***0.4750***	***0.0000***	***5.4681***	***0.5401***	***0.0000***	***5.0002***
SUCD5_BS	succinate dehydrogenase	fadh2[c] + (2) h[e] + mqn7[c] --> fad[c] + (2) h[c] + mql7[c]	Carbohydrates and related molecules	1.3.99.1	BG10353+ BG10352+ BG10351	-	-	-	***0.4353***	0.0000	5.5946	***0.3039***	***0.0000***	***3.4414***
SUCOAS	succinyl-CoA synthetase (ADP-forming)	[c] : atp + coa + succ <==> adp + pi + succoa	Carbohydrates and related molecules	6.2.1.5	BG12680, BG12681	***0.5542***	0.0000	0.0003	0.4612	0.0000	5.5116	0.5100	0.0000	5.0001

G_{max}: predicted maximum growth rate, P_{maxG}: predicted maximum 3-HP production rate at maximum growth rate, $P_{maxG/2}$: predicted maximum 3-HP production rate at half of the maximum growth rate, “-”: the deletion of corresponding reaction lead to no growth.

First line: Original glycerol dehydratase *dhaB1* from *K. pneumoniae*
Second line : codon optimized for *B. subtilis*
Third line : consensus

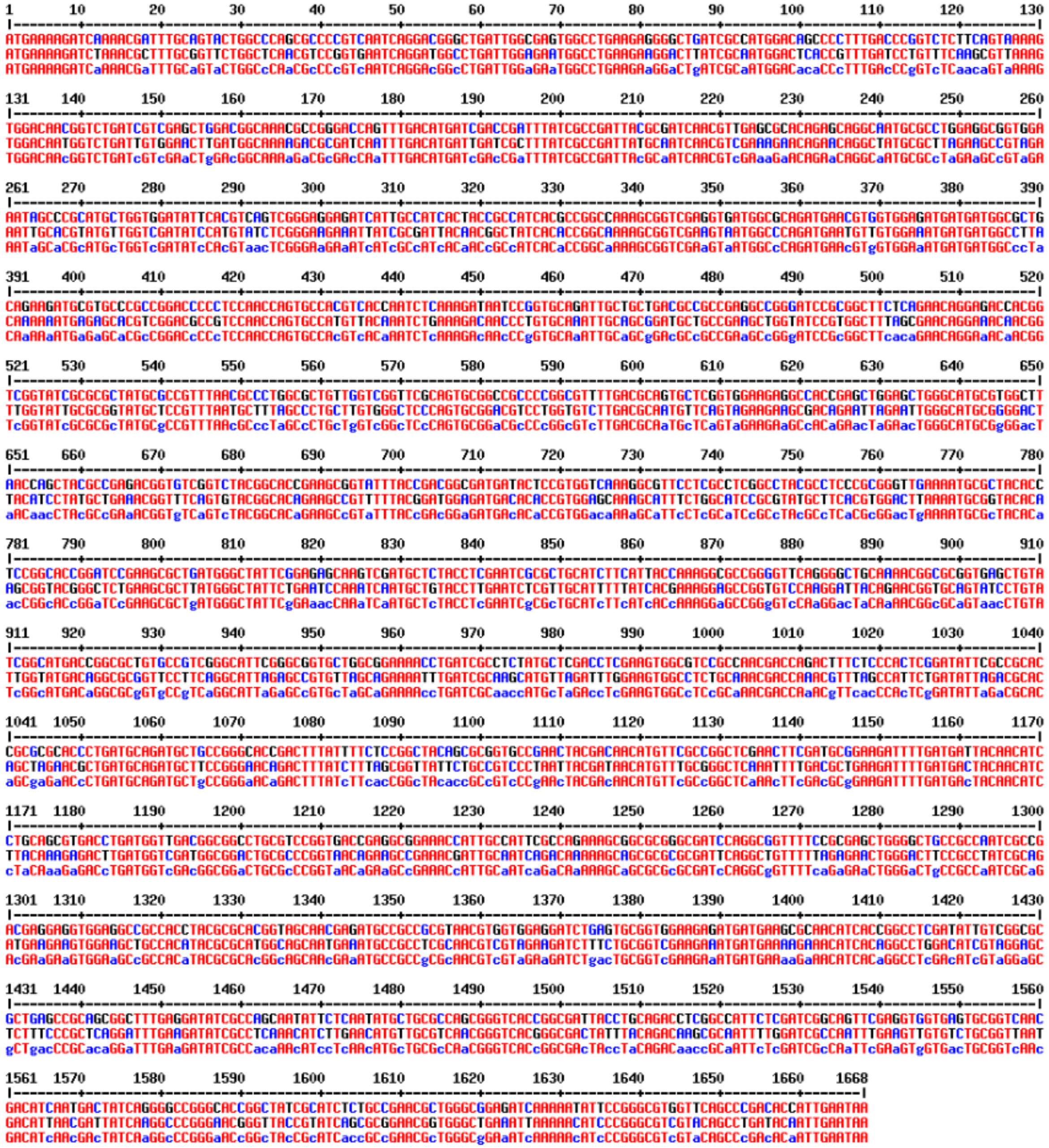

Supplementary Figure 1. Alignment of original sequence of the two step pathway for 3-HP production from glycerol (from *K. pneumoniae*) and the sequence codon optimized for *B. subtilis*

First line: Original glycerol dehydratase *dhaB2* subunit from *K. pneumoniae*
Second line : codon optimized for *B. subtilis*
Third line : consensus

```
1       10        20        30        40        50        60        70        80        90        100       110       120       130
GTGCAACAGACAACCCAAATTCAGCCCTCTTTTACCCTGAAAACCCGCGAGGGCGGGTAGCTTCTGCCGATGAACGCGCCGATGAAGTGGTGATCGGCGTCGGCCCTGCCTTCGATAAACACCAGCATC
ATGCAACAGACAACGCAAATCCAGCCGTCTTTTACACTTAAAACGCGCGAAGGCGGAGTTGCTAGCGCCGATGAACGTGCTGACGAAGTTGTGATCGGTGTGGGCCCTGCCTTTGATAAACATCAACATC
aTGCAACAGACAACcCAAATcCAGCCcTCTTTTACaCTgAAAACcCGCGAaGGCGGaGTaGCTaccGCCGATGAACGcGCcGAcGAAGTgGTGATCGGcGTcGGCCCTGCCTTcGATAAACAcCAaCATC

131     140       150       160       170       180       190       200       210       220       230       240       250       260
ACACTCTGATCGATATGCCCCATGGCGCGATCCTCAAAGAGCTGATTGCCGGGGTGGAAGAAGAGGGGCTTCACGCCCGGGTGGTGCGCATTCTGCGCACGTCCGACGTCTCCTTTATGGCCTGGGATGC
ATACATTGATCGACATGCCGCATGGCGCAATCCTGAAAGAACTTATTGCGGGTGTCGAAGAAGAAGGCTTACATGCAAGAGTCGTACGCATTTTGCGTACATCAGATGTTTCATTTATGGCGTGGGACGC
AcACacTGATCGAcATGCCcCATGGCGCaATCCTcAAAGAaCTgATTGCcGGgGTcGAAGAAGAaGGccTaCAcGCaaGaGTcGTaCGCATTcTGCGcACaTCaGAcGTcTCaTTTATGGCcTGGGAcGC

261     270       280       290       300       310       320       330       340       350       360       370       380       390
GGCCAACCTGAGCGGCTCGGGGATCGGCATCGGTATCCAGTCGAAGGGGACCACGGTCATCCATCAGCGCGATCTGCTGCCGCTCAGCAACCTGGAGCTGTTCTCCCAGGCGCCGCTGCTGACGCTGGAA
AGCGAATCTGTCAGGAAGCGGTATTGGCATCGGCATTCAATCAAAAGGCACAACGGTTATCCATCAGCGGGATCTGCTTCCTCTGTCTAACCTGGAACTGTTTTCACAAGCGCCGTTATTGACACTTGAA
aGCcAAcCTGacaGGaaccGGgATcGGCATCGGcATcCAaTCaAAaGGcACaACGGTcATCCATCAGCGcGATCTGCTgCCgCTcaccAACCTGGAaCTGTTcTCaCAaGCGCCGcTacTGACaCTgGAA

391     400       410       420       430       440       450       460       470       480       490       500       510       520
ACCTACCGGCAGATTGGCAAAAACGCCGCGCGCTATGCGCGCAAAGAGTCACCTTCGCCGGTGCCGGTGGTGAACGATCAGATGGTGCGGCCGAAATTTATGGCCAAAGCCGCGCTATTTCATATCAAAG
ACGTATAGACAGATTGGAAAAAATGCTGCCCGGTACGCAAGAAAAGAATCTCCGTCCCCTGTCCCGGTTGTGAACGATCAAATGGTACGCCCTAAATTTATGGCTAAAGCAGCGTTGTTTCATATCAAAG
ACcTAcaGaCAGATTGGaAAAAAcGCcGCcCGcTAcGCaaGaAAAGAaTCaCCgTCcCCgGTcCCGGTgGTGAACGATCAaATGGTaCGcCCgAAATTTATGGCcAAAGCaGCGcTaTTTCATATCAAAG

521     530       540       550       560       570       580  585
AGACCAAACATGTGGTGCAGGACGCCGAGCCCCTCACCCTGCACGTCGACTTAGTAAGGGAGTGA
AAACAAAACATGTCGTACAGGATGCCGAACCGTTAACGTTGCATGTTGACCTTGTGAGAGAATAA
AaACaAAACATGTcGTaCAGGAcGCCGAaCCccTaACccTGCAcGTcGACcTaGTaAGaGAaTaA
```

Supplementary Figure 1. Alignment of original sequence of the two step pathway for 3-HP production from glycerol (from *K. pneumoniae*) and the sequence codon optimized for *B. subtilis*

First line: Original glycerol dehydratase *dhaB3* subunit
from *K. pneumoniae*
Second line : codon optimized for *B. subtilis*
Third line : consensus

```
1        10        20        30        40        50        60        70        80        90        100       110       120       130
|--------+---------+---------+---------+---------+---------+---------+---------+---------+---------+---------+---------+---------|
ATGAGCGAGAAAACCATGCGCGTGCAGGATTATCCGTTAGCCACCCGCTGCCCGGAGCATATCCTGACGCCTACCGGCAAACCATTGACCGATATTACCCTCGAGAAGGTGCTCTCTGGCGAGGTGGGCC
ATGTCTGAAAAAACAATGAGAGTGCAAGATTATCCGCTGGCGACGCGCTGCCCTGAACATATTTTAACACCGACGGGTAAACCTTTGACAGATATCACGTTGGAAAAAGTTTTGTCTGGCGAAGTGGGAC
ATGaccGAaAAAACaATGaGaGTGCAaGATTATCCGcTaGCcACcCGCTGCCCgGAaCATATccTaACaCCgACcGGcAAACCaTTGACaGATATcACccTcGAaAAaGTgcTcTCTGGCGAaGTGGGaC

131      140       150       160       170       180       190       200       210       220       230       240       250       260
|--------+---------+---------+---------+---------+---------+---------+---------+---------+---------+---------+---------+---------|
CGCAGGATGTGCGGATCTCCTGCCAGACCCTTGAGTACCAGGCGCAGATTGCCGAGCAGATGCAGCGCCATGCGGTGGCGCGCAATTTCCGCCGCGCGGCGGAGCTTATCGCCATTCCTGACGAGCGCAT
CGCAAGACGTCCGTATTTCCTGTCAGACACTGGAATATCAAGCACAGATCGCGGAACAAATGCAGCGTCATGCAGTCGCGCGGAATTTTAGACGCGCAGCGGAACTTATTGCTATCCCGGATGAAAGAAT
CGCAaGAcGTcCGgATcTCCTGcCAGACaCTgGAaTAcCAaGCaCAGATcGCcGAaCAaATGCAGCGcCATGCaGTcGCGCGcAATTTcaGaCGCGCaGCGGAaCTTATcGCcATcCCgGAcGAaaGaAT

261      270       280       290       300       310       320       330       340       350       360       370       380       390
|--------+---------+---------+---------+---------+---------+---------+---------+---------+---------+---------+---------+---------|
TCTGGCTATCTATAACGCGCTGCGCCCGTTCCGCTCCTCGCAGGCGGAGCTGCTGGCGATCGCCGACGAGCTGGAGCACACCTGGCATGCGACAGTGAATGCCGCCTTTGTCCGGGAGTCGGCGGAAGTG
TTTAGCTATCTACAACGCCCTTCGTCCTTTTCGGTCAAGCCAGGCCGAACTGCTTGCTATTGCCGACGAATTAGAACATACATGGCATGCAACGGTAAATGCTGCCTTTGTTAGAGAATCAGCGGAAGTA
TcTaGCTATCTAcAACGCcCTgCGcCCgTTcCGcTCaaccCAGGCcGAaCTGCTgGCgATcGCCGACGAacTaGAaCAcACaTGGCATGCaACaGTaAATGCcGCCTTTGTcaGaGAaTCaGCGGAAGTa

391      400       410       420   426
|--------+---------+---------+-----|
TATCAGCAGCGGCATAAGCTGCGTAAAGGAAGCTAA
TACCAACAGAGACATAAATTGCGCAAAGGAAGCTAA
TAcCAaCAGaGaCATAAacTGCGcAAAGGAAGCTAA
```

Supplementary Figure 1. Alignment of original sequence of the two step pathway for 3-HP production from glycerol (from *K. pneumoniae*) and the sequence codon optimized for *B. subtilis*

First line: Original glycerol dehydratase *gdrA* subunit from *K. pneumoniae*
Second line : codon optimized for *B. subtilis*
Third line : consensus

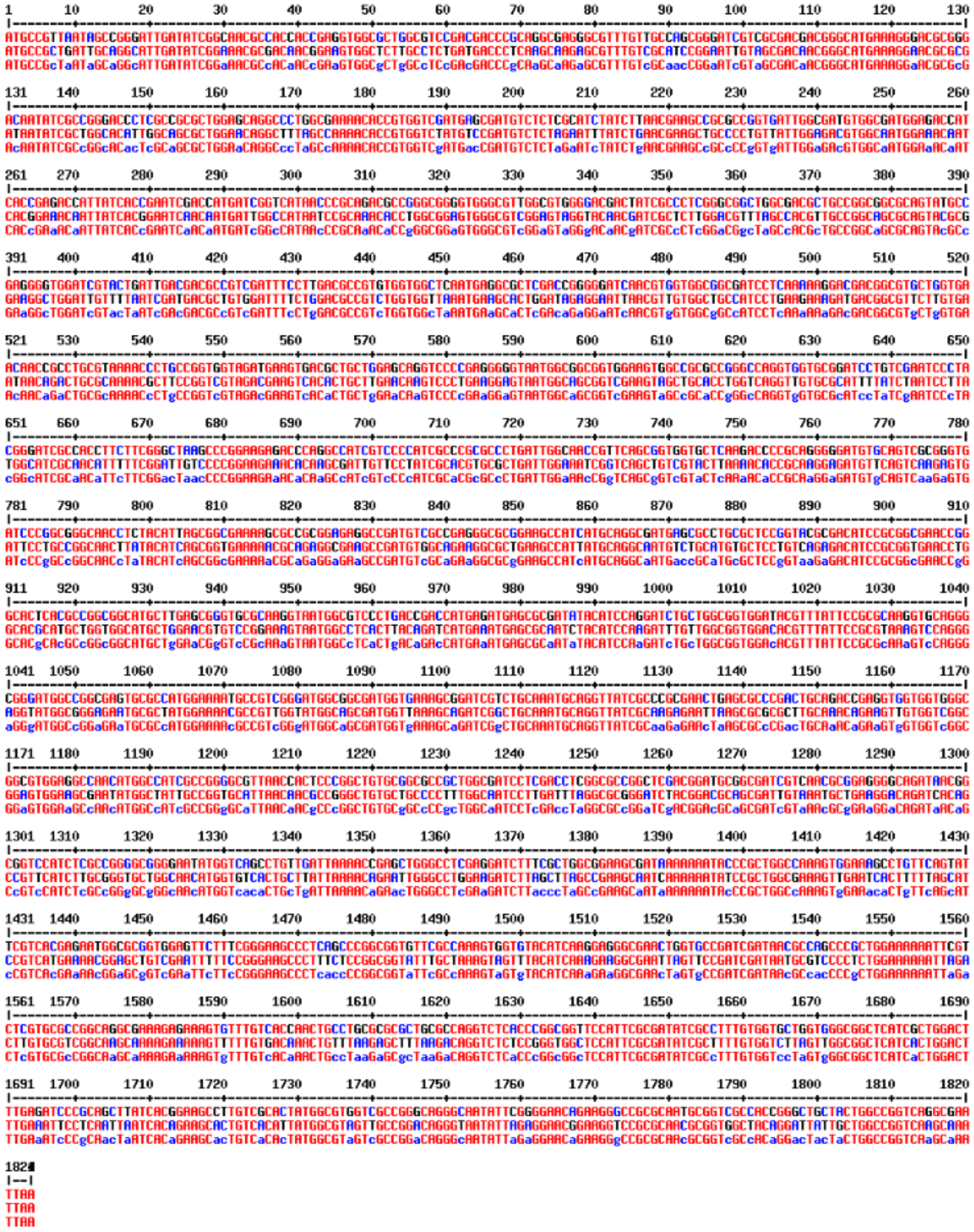

Supplementary Figure 1. Alignment of original sequence of the two step pathway for 3-HP production from glycerol (from *K. pneumoniae*) and the sequence codon optimized for B. subtilis

First line: Original glycerol dehydratase *gdrB* subunit
from *K. pneumoniae*
Second line : codon optimized for *B. subtilis*
Third line : consensus

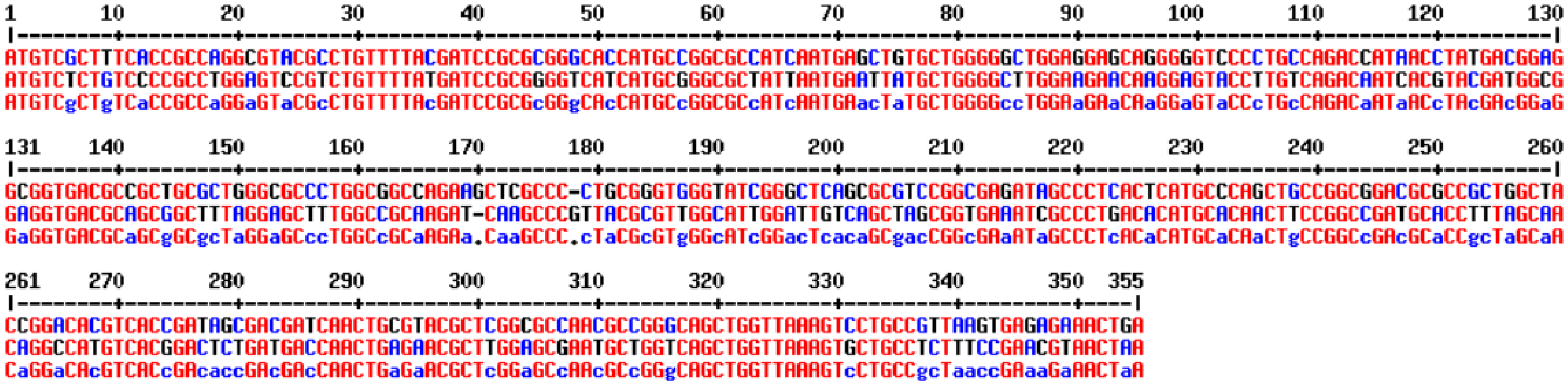

Supplementary Figure 1. Alignment of original sequence of the two step pathway for 3-HP production from glycerol (from *K. pneumoniae*) and the sequence codon optimized for B. subtilis

First line: Original *puuC* from *K. pneumoniae*
Second line : codon optimized for *B. subtilis*
Third line : consensus

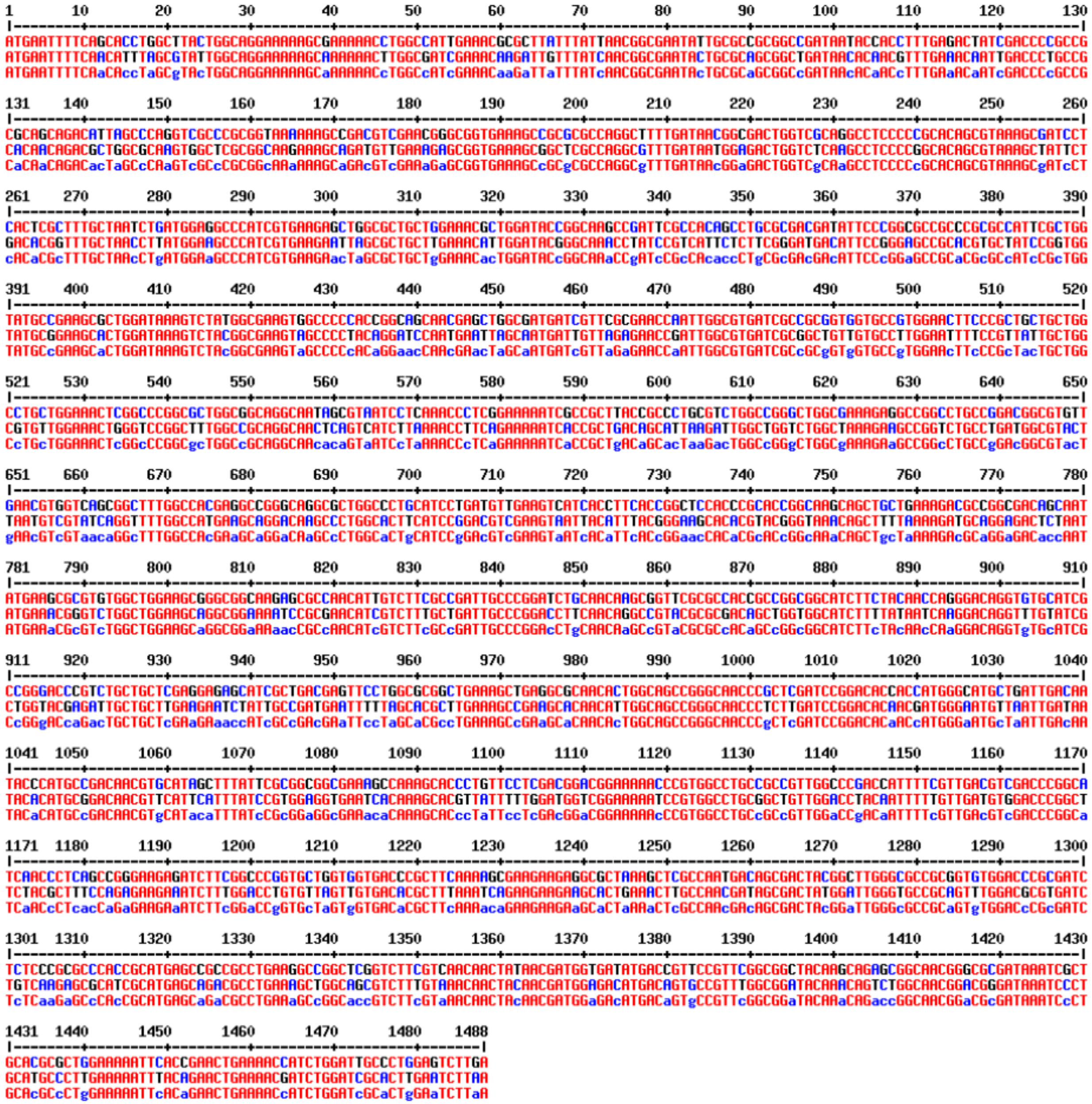

Supplementary Figure 1. Alignment of original sequence of the two step pathway for 3-HP production from glycerol (from *K. pneumoniae*) and the sequence codon optimized for B. subtilis

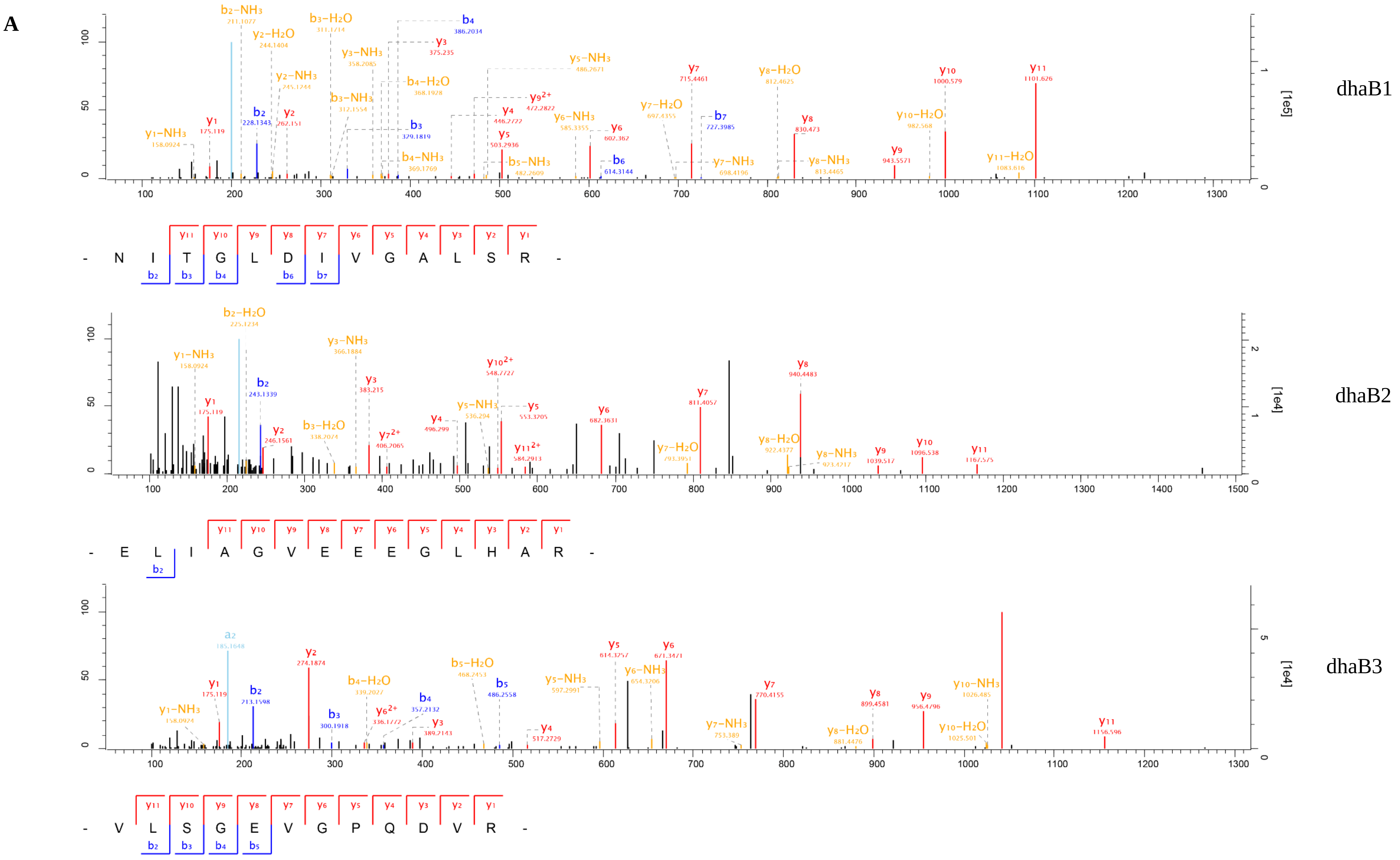

Supplementary Figure 2. MS/MS spectrum of the best identified peptides of glycerol dehydratase

B

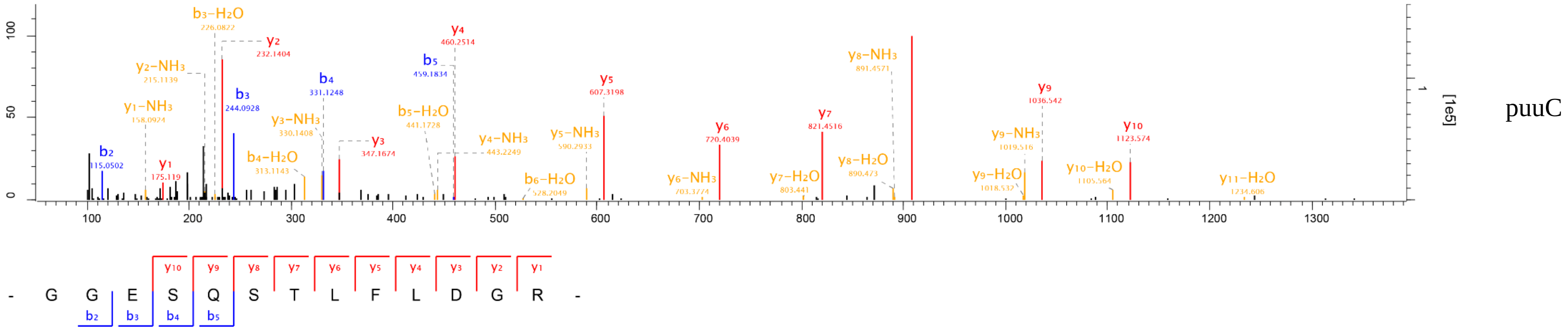

Supplementary Figure 2. MS/MS spectrum of the best identified peptides of aldehyde dehydrogenase

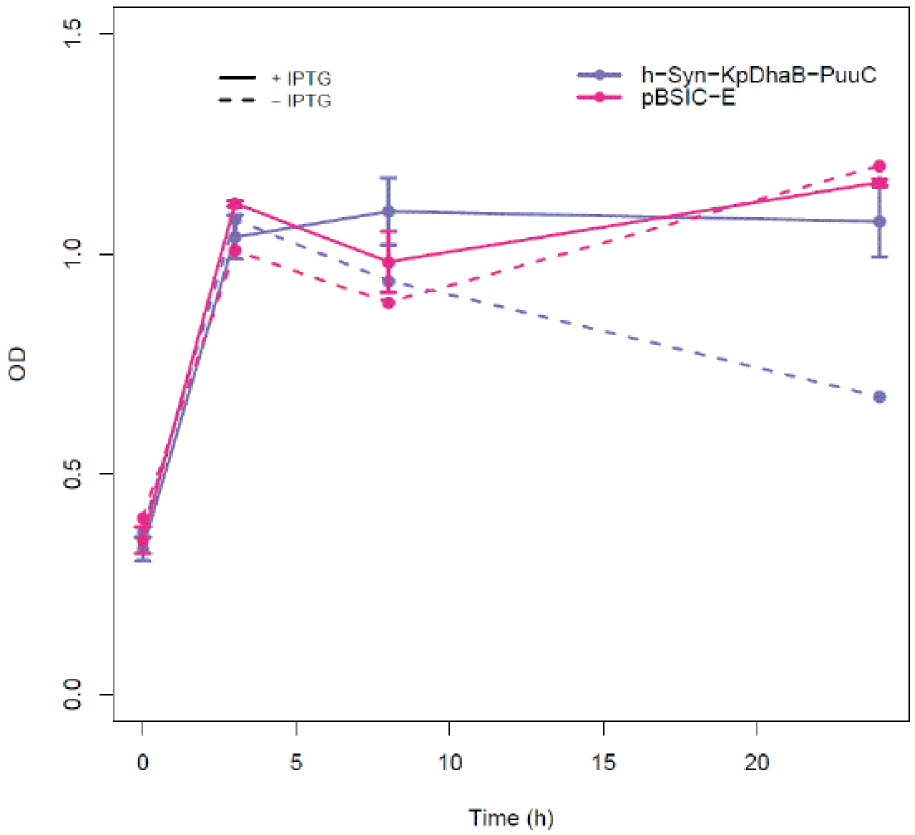

Supplementary Figure 3. Time course profile of the growth of h-syn-KpDhaB-PuuC recombinant strain and the WT (pBS1C-E) with or without IPTG induction.

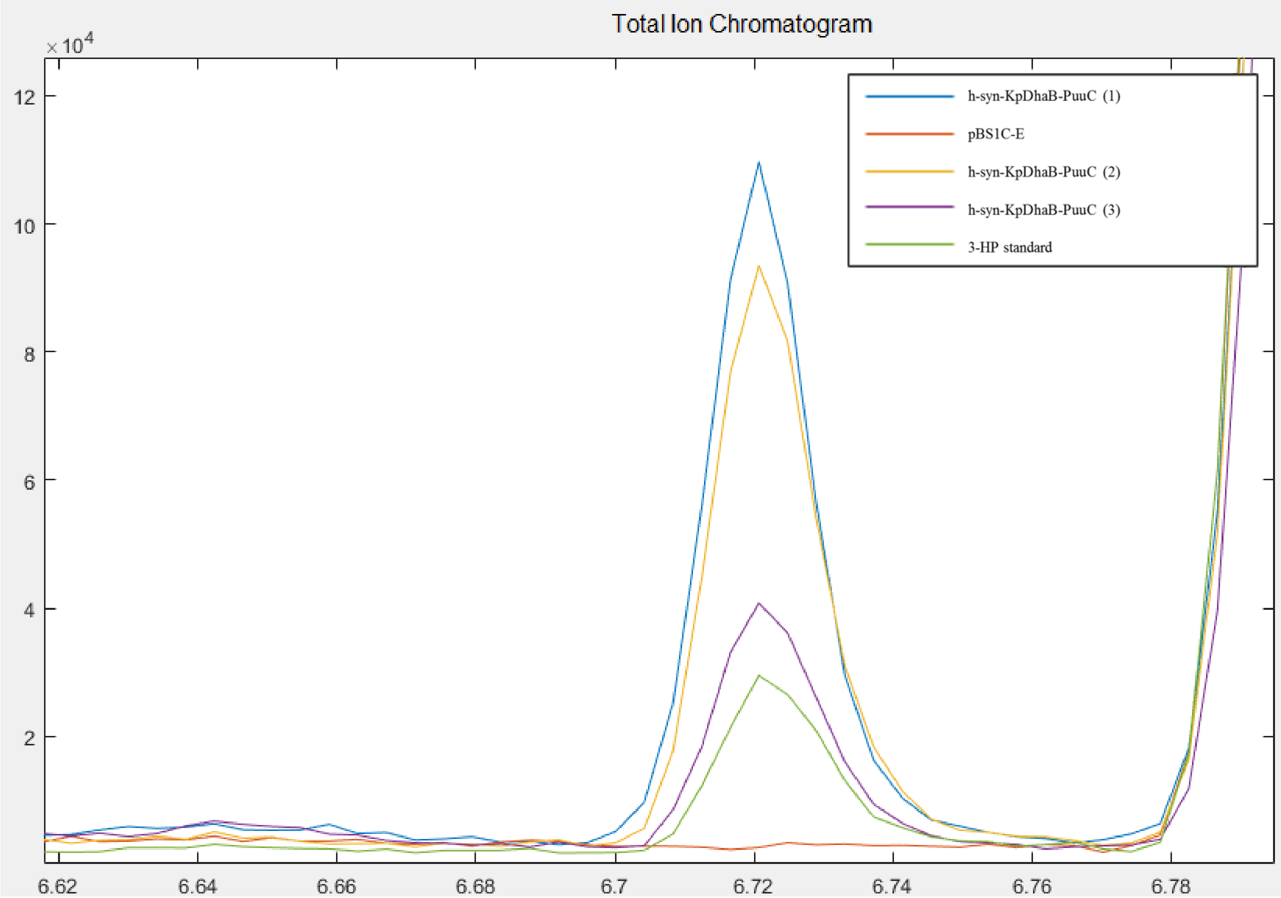

Supplementary figure 4. GC-MS results for h-syn-KpDhaB-PuuC recombinant strains and pBS1C-E control strain. 3-HP was detected in recombinant strain and not in the control strain.

Enzyme	Reaction	EC number
Glycerol dehydratase	Glycerol[c] → 3-HPA[c] + H_2O[c]	4.2.1.30
Aldehyde dehydrogenase	3-HPA[c] + NAD^+[c] + H_2O[c] → 3-HP[c] + NADH[c] + H^+[c]	1.2.1.3
3-HP transporter	3-HP[c] + H^+[c] + → 3-HP[e] + H^+[e]	-
3-HP exchange	3-HP[e] →	-

Supplementary Figure 5. Reactions added to the *Bacillus subtilis* GEM.

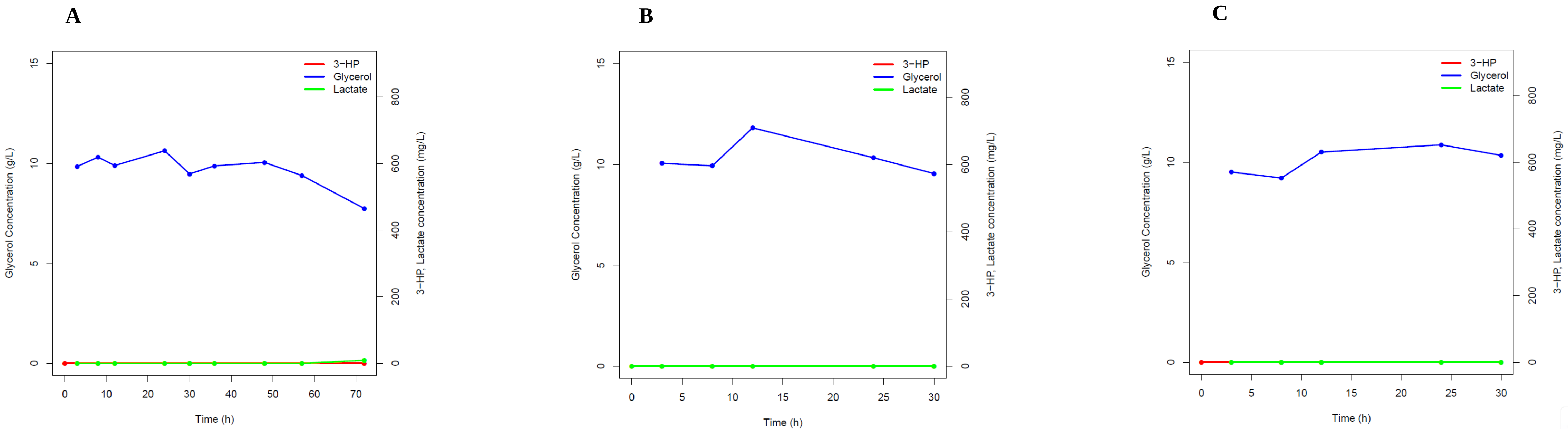

Supplementary Figure 6. Time course profile of the glycerol consumption, lactate and 3-HP production in *B. subtilis* strains. **(A)** the control strain with the empty plasmid (strain pBS1C-E-glpK), cultivated in M9 medium + glucose , induced by IPTG and supplemented with B12, and 12 g/L glycerol. (**B)** Recombinant strain knocked-out in glycerol kinase (*glpk*) overexpressing codon-optimized glycerol dehydratase and its activators (*dhaB123*, *gdrAB*) and the aldehyde dehydrogenase (*puuC*) from *K. pneumoniae* under control of phyperspank promoter (strain h-syn-KpDhaB-PuuC-glpK) cultivated in M9 medium + glucose , supplemented by B12 and 12 g/L glycerol, and (**C)** the control strain with the empty plasmid (strain pBS1C-E-glpK), cultivated in M9 medium + glucose , supplemented with B12 and 12 g/L glycerol. Glycerol consumption **(blue line)**, lactate production **(green line)**, 3-HP production (**red line**).

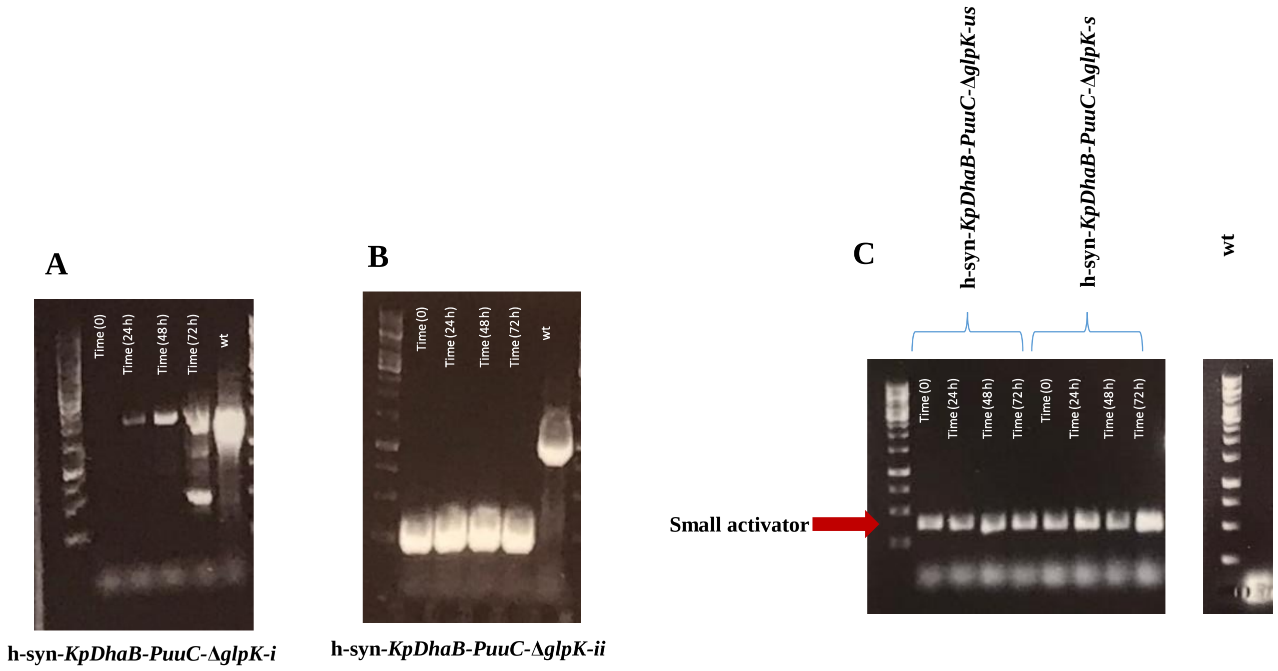

Supplementary Figure 7 . Stability test of genes using PCR in four different time points throughout the cultivation. (A) *glpK* knockout in h-syn-*KpDhaB-PuuC-ΔglpK*-i and (B) *glpK* knockout in h-syn-*KpDhaB-PuuC-ΔglpK*-ii (C) Synthetic pathway (amplifying *gdrB* gene) in h-syn-*KpDhaB-PuuC-ΔglpK*-I and h-syn-*KpDhaB-PuuC-ΔglpK*-ii
